# Association of Positive mHealth Engagement with Knowledge, Attitude, Practice, and Total KAP Among Patients with Multidrug-Resistant Tuberculosis

**DOI:** 10.3390/healthcare14111447

**Published:** 2026-05-23

**Authors:** Huy Le Ngoc, Giang Le Minh, Hoa Nguyen Binh, Luong Dinh Van

**Affiliations:** 1 School of Preventive Medicine and Public Health, Hanoi Medical University, Hanoi 100000, Vietnam; leminhgiang@hmu.edu.vn; 2 Vietnam National Lung Hospital, Hanoi 10000, Vietnam; nguyenbinhhoatb@yahoo.com (H.N.B.); dinhvanluong66@gmail.com (L.D.V.)

**Keywords:** multidrug-resistant tuberculosis, MDR-TB patients, mobile health, mHealth engagement, knowledge, attitudes, practices, digital health, treatment support, Vietnam

## Abstract

**Background:** Mobile health has been increasingly integrated into tuberculosis care to support patient education, communication, and treatment engagement. However, evidence remains limited regarding whether positive engagement with mHealth is associated with knowledge, attitudes, and practices among patients with multidrug-resistant tuberculosis. This study aimed to examine the association between positive mHealth engagement and knowledge, attitude, practice, and total KAP among patients with multidrug-resistant tuberculosis, and to evaluate the psychometric properties of the engagement score used as the primary exposure variable. **Methods:** A cross-sectional study was conducted among patients with multidrug-resistant tuberculosis. A positive mHealth engagement score was constructed from 12 mHealth-related items after harmonizing item directionality so that higher scores indicated more favorable engagement. The 12 items reflected five behavioural domains: intensity of use, ease and acceptability of use, functional engagement (communication with providers, access to health information, and perceived benefit for disease self-management), continuity of use, and barriers to sustained engagement. The composite score was computed as the mean of the 12 standardised items, with higher values indicating more positive engagement. Internal consistency was assessed using Cronbach’s alpha and corrected item–total correlations, and structural validity was explored using principal component analysis. Adjusted linear regression models were used to examine associations between the engagement score and Knowledge, Attitude, Practice, and total KAP scores, controlling for age, sex, and occupation. Sensitivity analyses were performed after excluding a poorly performing item, and tertile analyses were used to assess dose–response patterns. **Results:** The positive mHealth engagement score showed good internal consistency, with a Cronbach’s alpha of 0.852. One item demonstrated poor psychometric performance, and Cronbach’s alpha increased to 0.864 after its exclusion. The data were suitable for dimensionality assessment, with a Kaiser–Meyer–Olkin value of 0.870 and a significant Bartlett’s test. Principal component analysis identified a dominant first component explaining 43.29% of the total variance. Using the refined score, higher positive mHealth engagement was significantly associated with higher Knowledge scores (β = 2.06; 95% CI: 1.28–2.85; *p* < 0.001), higher Attitude scores (β = 4.68; 95% CI: 3.30–6.06; *p* < 0.001), and higher total KAP scores (β = 6.68; 95% CI: 4.62–8.74; *p* < 0.001), whereas no significant association was observed for the Practice score (β = −0.07; 95% CI: −0.63 to 0.49; *p* = 0.804). In tertile analyses, Knowledge, Attitude, and total KAP scores increased significantly across engagement levels, while Practice scores did not. **Conclusions:** Positive mHealth engagement was associated with better knowledge, attitudes, and overall KAP among patients with multidrug-resistant tuberculosis, but not with practice. These findings are associative; the cross-sectional design does not permit causal conclusions. The engagement score demonstrated good reliability and acceptable structural validity and may be a useful summary measure for evaluating patient interaction with mHealth interventions in tuberculosis care. Integrated strategies combining mHealth with clinical follow-up, adherence counseling, and structural support may be needed to translate informational and attitudinal gains into practice change.

## 1. Introduction

Multidrug-resistant tuberculosis (MDR-TB), defined as tuberculosis caused by Mycobacterium tuberculosis strains resistant to at least isoniazid and rifampicin, remains a major challenge to global tuberculosis control because treatment is prolonged, complex, and frequently accompanied by adverse events that can disrupt adherence and continuity of care. According to the WHO Global Tuberculosis Report 2025, an estimated 10.7 million people developed tuberculosis in 2024, with approximately 390,000 cases of MDR or rifampicin-resistant TB (MDR/RR-TB) and a global treatment success rate of approximately 68%. The WHO End TB Strategy explicitly recognises the potential of digital health technologies as enablers of improved treatment support and patient-centred care, particularly in high-burden settings such as Vietnam, which remains among the 30 countries with the highest MDR-TB burden worldwide [1,2]. Patients with multidrug-resistant tuberculosis often require sustained support throughout treatment, including counseling, symptom monitoring, and regular communication with healthcare providers [3]. In this context, mobile health (mHealth), defined as the use of mobile devices and wireless technology to support healthcare delivery, patient communication, and health behaviour change, has been increasingly explored as a way to strengthen patient-centred MDR-TB care and improve treatment support [4,5,6].

Positive patient engagement with mHealth may be especially relevant in multidrug-resistant tuberculosis care, where the burden of treatment extends over many months and patients must manage both the disease and the consequences of therapy [6,7]. When patients perceive digital health tools as useful, understandable, and supportive, such engagement may improve how they receive health information, how they understand treatment, and how they relate to care. These pathways are particularly relevant to knowledge, attitude, and practice outcomes, which are commonly used to assess patient-related dimensions of tuberculosis care. While knowledge and attitudes may respond relatively directly to digital communication and support, practice-related behaviors may also depend on broader structural, social, and treatment-related constraints [8].

Despite growing interest in digital health for tuberculosis, the literature has more often focused on adherence, feasibility, or implementation outcomes than on the relationship between patient engagement and knowledge, attitude, and practice. In addition, engagement is frequently treated as a simple intervention exposure rather than as a measurable patient-level construct with its own psychometric properties [4,9,10]. This is important because if engagement is measured using items that do not perform consistently, associations with downstream outcomes may be difficult to interpret. Evaluating the internal consistency and structural validity of a positive mHealth engagement score is therefore an important step before using it as an analytical variable.

Engagement with digital health interventions is increasingly recognised as a multidimensional construct encompassing the frequency, depth, and quality of patient interaction with technology, as well as the perceived value and subjective experience of that interaction. Theoretical frameworks of digital engagement distinguish between behavioural engagement (how often and for how long a patient uses the tool), functional engagement (how actively and purposefully the patient uses the available features), and experiential engagement (whether the patient perceives the tool as useful, comprehensible, and supportive) [11]. This distinction matters because patients who use an application frequently but derive little perceived benefit may differ substantially in health-related outcomes from patients who use the application less often but report high perceived value and active interaction with clinical features. Treating engagement as a patient-level construct with its own psychometric properties—rather than as a simple binary measure of whether a patient used the application—allows more rigorous evaluation of its role in predicting knowledge, attitudes, and practices.

Despite the growing adoption of mHealth tools in MDR-TB care, patient engagement with these interventions varies considerably—some patients use the application frequently and report high perceived value, while others use it rarely or discontinue use before treatment is complete. This variation in engagement is clinically meaningful: if engagement is associated with better knowledge, more positive treatment attitudes, or more adherent practice, then identifying and addressing low engagement becomes an important target for care improvement. However, to examine this association rigorously, engagement must first be measured as a patient-level construct with defined items, verified internal consistency, and confirmed structural validity. A validated engagement score is therefore not an end in itself, but a prerequisite for any meaningful inference about the relationship between patient interaction with mHealth and health-related outcomes.

The KAP framework—encompassing knowledge, attitudes, and practices—provides a well-established structure for assessing patient-related dimensions of tuberculosis care. In the context of MDR-TB, this framework is particularly appropriate because treatment is knowledge-intensive (patients must understand adherence importance, treatment duration, adverse effects, and their management), attitudes are central determinants of whether patients remain engaged across many months of therapy, and practice represents the behavioural endpoint toward which knowledge and attitudinal improvement should ultimately translate. Crucially, separating knowledge, attitudes, and practices as distinct outcome domains allows a more nuanced examination of where mHealth engagement has its effects: digital information delivery may influence knowledge directly, attitudinal change may follow repeated positive interactions, and practice change may additionally depend on structural and social factors beyond the application itself.

This study was conducted within the V-SMART trial, a randomised controlled trial of a smartphone-based mHealth platform developed to support MDR-TB care in Vietnam, with a focus on treatment communication and adverse event management. Because all participants in the present analysis were enrolled in the intervention arm and had access to the same application, the dataset provides a natural setting in which to observe variation in patient engagement arising from differences in patients’ willingness, ability, and motivation to interact with the application—rather than from differences in access. This variation in engagement, and its potential relationship with KAP outcomes, forms the basis of the present study’s hypothesis [12,13]. More recent work from the same project has also highlighted challenges in sustaining user engagement with the application over time, reinforcing the importance of understanding how patients interact with the intervention rather than assuming that access alone is sufficient. Therefore, the present study aimed to evaluate the psychometric properties of a positive mHealth engagement score and to examine its association with knowledge, attitude, practice, and total KAP among patients with multidrug-resistant tuberculosis. We further assessed the robustness of these associations in sensitivity analyses and explored whether KAP outcomes showed a dose–response pattern across levels of engagement. To our knowledge, this is the first study to psychometrically validate a composite mHealth engagement score and examine its association with knowledge, attitudes, and practices as distinct outcome domains in an MDR-TB population in Vietnam, contributing to the growing evidence base for digital health tools in patient-centred tuberculosis care.

## 2. Materials and Methods

### 2.1. Study Design and Setting

This cross-sectional study was conducted among patients with multidrug-resistant tuberculosis (MDR-TB) in Vietnam, within the broader context of the V-SMART mHealth-supported care programme. Participants were exposed to a smartphone-based intervention designed to support treatment communication and monitoring, and the present study examined the association between positive mHealth engagement and knowledge, attitude, and practice (KAP) outcomes.

A cross-sectional design was selected as appropriate for two complementary aims: (1) evaluating the psychometric properties of the engagement score, for which a single time-point observation is sufficient to assess internal consistency and structural validity; and (2) estimating associations between engagement and KAP outcomes as a basis for hypothesis generation. The inherent limitation of this design—its inability to establish temporal precedence or causal direction—is acknowledged explicitly and is discussed in the Limitations section. Future longitudinal or cohort studies would be needed to evaluate whether changes in engagement precede and predict changes in KAP outcomes over time.

### 2.2. Study Population and Participants

The study population included patients diagnosed with multidrug-resistant tuberculosis who were enrolled in the intervention arm of the V-SMART trial and had available data on mHealth engagement and KAP-related measures. Participants were enrolled through consecutive sampling of all eligible MDR-TB patients at participating sites who met the inclusion criteria and provided written informed consent during the study period.

A total of 278 patients contributed complete data on the KAP outcome variables and were included in all descriptive and outcome analyses. Of these, 231 (83.1%) had complete responses across all 12 engagement items and were included in the psychometric analyses and adjusted regression models. The remaining 47 participants (16.9%) had missing responses on one or more engagement items and were excluded from the engagement-based analyses under a complete-case approach. Missingness in the engagement items was predominantly structural rather than random: the discontinuation sub-items (Thongtin11.1–11.4) were conditional questions applicable only to patients who had stopped using the application before treatment completion; patients who had used the application continuously throughout treatment were not presented with these items and therefore had no data on them. Baseline demographic and treatment characteristics of participants with complete engagement data (n = 231) were broadly similar to those of excluded participants (n = 47), supporting the validity of the complete-case analytic approach.

### 2.3. Study Variables

The main explanatory variable was positive mHealth engagement, derived from patient responses to a set of mHealth-related questionnaire items reflecting favorable perceptions and experiences of the intervention. Item directionality was harmonized before score construction so that higher values consistently indicated more positive engagement. The items were then combined into a composite engagement score.

The study outcomes were Knowledge score, Attitude score, Practice score, and Total KAP score, each analyzed as a continuous variable. Multivariable models adjusted for age, sex, and occupation as potential confounders.

Development and Psychometric Evaluation of the Positive mHealth Engagement Score.

A composite positive mHealth engagement score was constructed from 12 questionnaire items. Internal consistency was assessed using Cronbach’s alpha and corrected item–total correlations. Items with poor psychometric contribution were identified based on low or negative item–total correlations and by examining changes in Cronbach’s alpha after item deletion.

To explore structural validity, principal component analysis (PCA) was performed after confirming the suitability of the data. Sampling adequacy was evaluated using the Kaiser–Meyer–Olkin statistic, and the correlation structure was assessed using Bartlett’s test of sphericity. Because one item demonstrated poor psychometric performance, a sensitivity analysis was conducted using a refined engagement score that excluded this item.

### 2.4. Positive mHealth Engagement Score

The main explanatory variable was the positive mHealth engagement score, derived from 12 patient-reported items collected within the V-SMART intervention. Items were organised across five behavioural domains:

Domain A—Intensity of use (1 item): frequency of application use during the treatment period (Thongtin2), assessed on an ordinal scale from daily to never.

Domain B—Ease and acceptability (1 item): perceived ease of using the application (Thongtin3), assessed on an ordinal scale from very easy to very difficult.

Domain C—Functional engagement (4 items): use of in-app communication with doctors or healthcare staff (Thongtin4), perceived benefit for understanding disease and medication use (Thongtin5), access to health information and educational articles via the application (Thongtin6), and perceived benefit for disease self-management and wellbeing (Thongtin7).

Domain D—Continuity and disruption (2 items): number of times the application was reinstalled during treatment (Thongtin8), and the longest continuous period of application non-use during treatment (Thongtin10).

Domain E—Discontinuation barriers (4 items): four binary items (0 = No, 1 = Yes) reflecting main reasons for stopping application use before treatment completion, including device change or application malfunction (Thongtin11.1), changing perceived need (Thongtin11.2), feeling the application was no longer necessary (Thongtin11.3), and other reasons (Thongtin11.4).

Items in Domains D and E reflected disruptive or negative engagement experiences. These items were reverse-scored prior to composite construction using the formula x_1_^eu^ = max + min − x, where max and min are the observed maximum and minimum values of each item, so that higher values consistently indicated more positive engagement across all 12 items. Each item was then standardised to a z-score (z = [x − mean]/SD) using that item’s own mean and standard deviation. The composite positive mHealth engagement score was computed as the arithmetic mean of the 12 standardised items. This mean-of-z-scores approach produces a composite centred near zero with a standard deviation of approximately 0.5 (not 1.0, because averaging across items reduces variance), which is consistent with the observed distribution in this sample (mean = 0.11, SD = 0.50, range = −1.39 to 1.47). All regression coefficients reported in this paper therefore represent the change in KAP outcome score associated with a one-unit increase in the engagement score, which corresponds to approximately two standard deviations on this scale.

### 2.5. KAP Outcome Variables

The study outcomes were Knowledge score, Attitude score, Practice score, and Total KAP score, each analysed as a continuous variable. The KAP instrument was developed specifically for the V-SMART trial, drawing on the V-SMART protocol, established MDR-TB treatment guidelines, and existing validated KAP instruments for tuberculosis populations [14,15]. Content validity was established through expert review by a panel of clinicians and public health researchers. The instrument was piloted in 30 MDR-TB patients not included in the main analysis; items with ambiguous wording or cultural inappropriateness were revised accordingly.

Knowledge domain: 16 scored items covering the definition of MDR-TB, modes of transmission, risk factors, treatment principles, treatment duration, common adverse effects, and their management. Each item had one correct response, scored 1 point, yielding a maximum Knowledge score of 16.

Attitude domain: 11 items assessed on a 5-point Likert scale (0 = strongly disagree to 4 = strongly agree), covering beliefs about treatment importance, adherence, side-effect reporting, and support-seeking behaviour. Three negatively worded items (reflecting beliefs that patients could self-manage side effects, should double doses after a missed dose, and did not need to report co-medications) were reverse-scored so that higher values consistently reflected more positive treatment-related attitudes. The maximum Attitude score was 44.

Practice domain: 12 items assessing self-reported treatment-related behaviours, including medication adherence, dose omission frequency, confidence in adherence, follow-up attendance, side-effect management, and communication with healthcare providers. A weighted scoring system with skip logic was applied: patients who reported never forgetting to take their medication received full credit for the adherence sub-items they were not asked to complete (maximum 10 points for the adherence block), and patients who reported attending all scheduled check-ups received full credit for the check-up sub-items (maximum 4 points). The maximum Practice score was 21.

A Total KAP score was computed by summing the three domain scores (theoretical maximum = 81; observed maximum in this sample = 78). Higher scores on all domains and on the Total KAP score indicate more favourable knowledge, attitudes, and practices. Internal consistency was good: Cronbach’s α = 0.78 for the knowledge domain, 0.85 for the attitude domain, and 0.85 for the total KAP score, all exceeding the conventional threshold of 0.70. Cronbach’s α was not computed for the practice domain due to its mixed item types and skip logic structure, which violate the assumptions of the standard α model. Multivariable models adjusted for age, sex, and occupation as potential confounders.

### 2.6. Development and Psychometric Evaluation of the Positive mHealth Engagement Score

A composite positive mHealth engagement score was constructed from the 12 items. Internal consistency was assessed using Cronbach’s alpha and corrected item–total correlations. Items with poor psychometric contribution were identified based on low or negative corrected item–total correlations and by examining changes in Cronbach’s alpha after item deletion.

One item—Thongtin11.1, capturing whether device change or application malfunction was the main reason for stopping application use before treatment completion—demonstrated poor psychometric performance, with a corrected item–total correlation of −0.102, well below the conventional threshold of 0.30 and the only item with a negative value. This item reflects externally driven technical disruptions outside the patient’s control rather than patient-driven engagement behaviour, explaining its poor coherence with the remaining items. Cronbach’s alpha increased from 0.852 to 0.864 following its exclusion. The refined 11-item score (excluding Thongtin11.1) was used in all primary regression and tertile analyses reported in this paper.

To explore structural validity, principal component analysis (PCA) was performed after confirming data suitability. Sampling adequacy was evaluated using the Kaiser–Meyer–Olkin (KMO) statistic, and the correlation structure was assessed using Bartlett’s test of sphericity. The first principal component had an eigenvalue of 5.218, explaining 43.29% of the total variance. Two additional components had eigenvalues greater than 1, indicating some degree of multidimensionality. While the scale is treated as essentially unidimensional for the purpose of producing a composite score—given the substantially dominant first component and the coherent theoretical framework linking all items to a single engagement construct—we acknowledge that the five conceptual domains may represent partially distinct subdomains that future confirmatory factor analysis with larger samples should formally evaluate.

### 2.7. Statistical Analysis

Descriptive and inferential analyses were performed to examine the association between positive mHealth engagement and KAP outcomes. The engagement score was first analyzed as a continuous variable in multivariable linear regression models, with Knowledge, Attitude, Practice, and Total KAP entered separately as dependent variables. All models adjusted for age, sex, and occupation.

To assess robustness, the adjusted regression analyses were repeated using the refined 11-item engagement score after exclusion of the poorly performing item. Regression coefficients (β), standardised regression coefficients (β*), 95% confidence intervals, and *p*-values were reported. Standardised coefficients were computed as β* = β × (SD_x_/SDᵧ), where SD_x_ is the standard deviation of the engagement score (0.50) and SDᵧ is the standard deviation of each KAP outcome, to allow comparison of effect magnitudes across domains.

To evaluate dose–response patterns, the refined engagement score was categorised into tertiles representing low, moderate, and high engagement. Mean KAP scores were compared across tertiles, and linear trend analyses were used to assess dose–response patterns across engagement levels. A two-sided *p*-value of <0.05 was considered statistically significant.

The primary analytic sample of 278 participants was determined by the enrolment capacity of the V-SMART intervention; no prospective power calculation was conducted. Post hoc power calculations confirmed that the analytic sample (n = 231 with complete engagement data) provided greater than 80% power to detect associations of the magnitude observed (Knowledge β = 2.06, Attitude β = 4.68, Total KAP β = 6.68) at α = 0.05, given the observed standard deviations of the engagement score (SD = 0.50) and outcome variables. The null association with Practice (β = −0.07; 95% CI: −0.63 to 0.49) was estimated with sufficient precision to conclude the absence of a clinically meaningful association. Prior to modelling, regression assumptions were verified: normality of residuals was assessed by inspection of Q–Q plots, linearity was evaluated using component-plus-residual plots, and homoscedasticity was assessed using residual-versus-fitted plots. No major violations were identified.

### 2.8. Ethical Considerations

Ethical approval for this study was granted by the University of Sydney Human Research Ethics Committee (2019/676); the Scientific Committee of the Ministry of Science and Technology, Vietnam (08/QD-HDQL-NAFOSTED); and the Institutional Review Board of the National Lung Hospital, Vietnam (13/19/CT-HDDD). Additional approval was obtained from the Vietnam National Lung Hospital Ethics Committee (VNLH-2023-01). All participants provided informed consent prior to enrollment, and the study analyzed de-identified patient data to ensure strict confidentiality and adherence to the approved research protocol.

## 3. Results

The participants had a mean age of 42.96 years with a standard deviation of 13.64 years, and the median age was 41.0 years with an interquartile range of 32.0 to 54.0 years. Most participants were male (66.2%), while 33.8% were female. In terms of occupation, the largest group was freelance or informal laborers (55.0%), followed by other occupations (26.3%), retired participants (12.2%), and office workers (6.5%). Regarding treatment history, 78.4% were in their first MDR-TB treatment course, 20.5% were in their second treatment course, and 1.1% were in their third treatment course.

### 3.1. Psychometric Properties of the Positive mHealth Engagement Score

The positive mHealth engagement scale demonstrated good internal consistency. The 12-item scale had a Cronbach’s alpha of 0.852, based on 231 complete cases, indicating that the items showed acceptable to strong coherence as a composite measure of positive engagement.

The data were also suitable for dimensionality assessment. The Kaiser–Meyer–Olkin value was 0.870, suggesting good sampling adequacy, and Bartlett’s test of sphericity supported sufficient inter-item correlation for factor-based assessment.

(χ^2^ = 1351.31, *df* = 66). Together, these findings support the use of the engagement items as a meaningful scale for subsequent analysis.

### 3.2. Association Between Positive mHealth Engagement and KAP Outcomes

In the multivariable linear regression analyses, positive mHealth engagement was significantly associated with better knowledge, attitude, and total KAP, but not with practice. Interpreting the score in the positive direction, higher engagement was associated with better KAP performance.

Using the refined score, per one-unit increase in the positive mHealth engagement score, higher positive mHealth engagement was significantly associated with higher Knowledge, Attitude, and Total KAP scores. Specifically, each one-unit increase in the positive mHealth engagement score was associated with a 2.06-point increase in Knowledge score (95%CI: 1.28 to 2.85, *p* < 0.001), a 4.68-point increase in Attitude score (95%CI: 3.30 to 6.06, *p* < 0.001), and a 6.68-point increase in Total KAP score (95%CI: 4.62 to 8.74, *p* < 0.001). In contrast, no significant association was observed between positive mHealth engagement and Practice score (β = −0.07, 95%CI: −0.63 to 0.49, *p* = 0.804).

A clear gradient was observed when participants were categorized into tertiles of positive mHealth engagement. Mean Knowledge scores increased from 8.42 in the low-engagement group to 10.67 in the moderate-engagement group and 11.69 in the high-engagement group (*p* < 0.001). A similar pattern was found for Attitude scores, which rose from 32.53 to 35.67 and 39.21, respectively (*p* < 0.001). Total KAP scores also increased steadily across tertiles, from 59.37 to 64.67 and 69.13 (*p* < 0.001).

By contrast, Practice scores remained relatively stable across engagement levels, with mean values of 18.42, 18.33, and 18.23 in the low-, moderate-, and high-engagement groups, respectively (*p* = 0.847).

Adjusted trend analyses confirmed these dose–response findings. Each increase in engagement tertile was associated with a 1.58-point increase in Knowledge score (*p* < 0.001), a 3.17-point increase in Attitude score (*p* < 0.001), and a 4.71-point increase in Total KAP score (*p* < 0.001). No significant linear trend was observed for Practice score (*β* = −0.045, *p* = 0.792).

Table 1 presents the distribution of the main study variables. The mean Knowledge score was 9.95 ± 3.39, with a median of 11.0 (*IQR*: 8.0–13.0). The mean Attitude score was 35.63 ± 5.99, and the median was 35.5 (*IQR*: 31.0–41.0). The mean Practice score was 18.25 ± 2.37, with a median of 19.0 (*IQR*: 18.0–20.0). The mean Total KAP score was 63.83 ± 8.40, and the median was 64.0 (*IQR*: 57.0–71.0). Among the 231 participants with complete engagement data, the mean positive mHealth engagement score was 0.11 ± 0.50, with a median of 0.03 (IQR:−0.31 to 0.55).

Table 2 shows the psychometric properties of the 12-item positive mHealth engagement scale.

The positive mHealth engagement scale demonstrated good internal consistency. The 12-item scale had a Cronbach’s alpha of 0.852, based on 231 complete cases, indicating that the items showed acceptable to strong coherence as a composite measure of positive engagement. One item (Thongtin11.1) demonstrated poor psychometric performance, with a corrected item–total correlation of −0.102; Cronbach’s alpha improved from 0.852 to 0.864 following its exclusion. The refined 11-item score was used in all primary analyses.

The data were also suitable for dimensionality assessment. The Kaiser–Meyer–Olkin value was 0.870, suggesting good sampling adequacy, and Bartlett’s test of sphericity supported sufficient inter-item correlation for factor-based assessment (χ^2^ = 1351.31, df = 66, *p* < 0.001). The first principal component had an eigenvalue of 5.218, explaining 43.29% of the total variance. Two additional components had eigenvalues greater than 1, indicating some degree of multidimensionality; this is acknowledged as a limitation. Together, these findings support the use of the engagement items as a meaningful scale for subsequent analysis.

After confirming the psychometric adequacy of the positive mHealth engagement scale, regression analyses were conducted to assess its association with KAP outcomes. As shown in Table 3, higher positive mHealth engagement was significantly associated with higher Knowledge, Attitude, and Total KAP scores. Specifically, each one-unit increase in the positive mHealth engagement score was associated with a 2.06-point increase in Knowledge score (95% CI: 1.28 to 2.85, *p* < 0.001), a 4.68-point increase in Attitude score (95% CI: 3.30 to 6.06, *p* < 0.001), and a 6.68-point increase in Total KAP score (95% CI: 4.62 to 8.74, *p* < 0.001). In contrast, no significant association was observed between positive mHealth engagement and Practice score (*β* = −0.07, 95% CI: −0.63 to 0.49, *p* = 0.804).

The clinical significance of these associations warrants consideration alongside their statistical significance. A 2.06-point increase in Knowledge score per unit of engagement corresponds to approximately 12.9% of the maximum Knowledge score (range 0–16), equivalent to answering approximately 2–3 additional knowledge items correctly—a meaningful improvement for patients managing a complex, long-duration treatment. A 4.68-point increase in Attitude score corresponds to approximately 10.6% of the maximum Attitude score (range 22–44), representing a meaningful shift toward more positive treatment-related beliefs. A 6.68-point increase in Total KAP score corresponds to approximately 8.2% of the theoretical maximum (81 points). The absence of a meaningful association with Practice (β = −0.07; 95% CI: −0.63 to 0.49) suggests that barriers to practice improvement in MDR-TB care lie largely outside the reach of digital engagement alone.

These findings indicate that positive engagement with mHealth was more strongly related to knowledge and attitudes than to behavioral practice.

To facilitate interpretation of the regression findings, participants were categorized into low, moderate, and high tertiles of positive mHealth engagement. As presented in Table 4, mean Knowledge, Attitude, and Total KAP scores increased progressively across engagement tertiles. Mean Knowledge scores rose from 8.42 in the low-engagement group to 10.67 in the moderate-engagement group and 11.69 in the high-engagement group. Mean Attitude scores similarly increased from 32.53 to 35.67 and 39.21, respectively. Mean Total KAP scores increased from 59.37 to 64.67 and 69.13 across the three tertiles.

By contrast, Practice scores showed minimal variation across groups, with means of 18.42, 18.33, and 18.23 in the low-, moderate-, and high-engagement tertiles, respectively, and the between-group difference was not statistically significant (*p* = 0.847).

**Figure 1:**
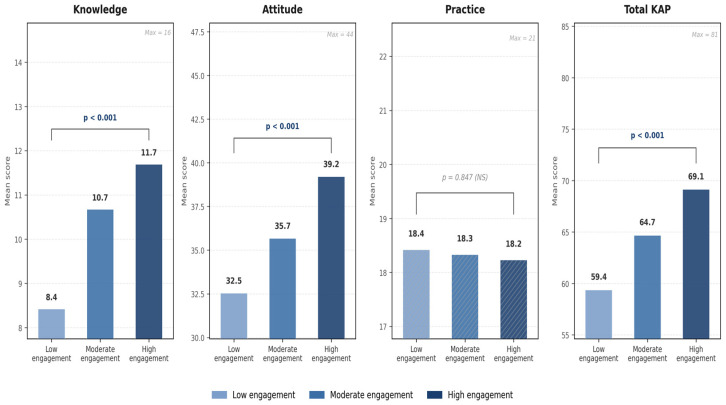
Mean KAP domain scores stratified by tertiles of positive mHealth engagement (low, moderate, high).

Figure 1 presents the mean KAP domain scores stratified by engagement tertile. The progressive increase across tertiles for Knowledge, Attitude, and Total KAP scores, contrasted with the flat trajectory for Practice, is visually apparent. This figure complements the regression findings by illustrating the dose–response gradient across engagement levels.

Overall, the tertile-based analysis was consistent with the regression results, showing a clear positive gradient for knowledge and attitudes, but not for practice. Standardised regression coefficients (Table 3) further confirmed that the associations with Attitude (β* = 0.391) and Total KAP (β* = 0.398) were the strongest, followed by Knowledge (β* = 0.304), while the association with Practice was negligible (β* = −0.015).

## 4. Discussion

It is important to note at the outset that all findings in this study are associations rather than causal effects. The cross-sectional design cannot establish whether higher positive mHealth engagement led to better knowledge and attitudes, or whether patients with better baseline knowledge and more positive attitudes were more likely to engage actively with the application. Both directions are plausible, and the present data cannot distinguish between them. Accordingly, associative language is used throughout this discussion.

This study evaluated the psychometric performance of the positive mHealth engagement scale and examined its association with knowledge, attitudes, and practices among patients with multidrug-resistant tuberculosis who were using an mHealth application designed to support medication adherence and adverse event reporting [6,12]. Two main findings emerged. First, the positive mHealth engagement scale demonstrated good internal consistency and acceptable psychometric adequacy, supporting its use in this study population. Second, higher positive mHealth engagement was significantly associated with better knowledge and more favourable attitudes, whereas no statistically significant association was observed for practice. This pattern was consistent across both regression and tertile-based analyses.

The psychometric findings are important because they support the validity of using the engagement score as a meaningful exposure variable in this setting. The 12-item scale showed good reliability, with a Cronbach’s alpha of 0.852, and the sampling adequacy statistics also supported its internal coherence. In practical terms, this suggests that the scale captured a reasonably stable construct reflecting how positively and actively patients engaged with the mHealth application. This is particularly relevant in MDR-TB care, where digital tools are increasingly used not only to deliver information, but also to support long-term treatment monitoring, adherence, and communication about adverse events [14,15].

It is important to clarify what “higher positive mHealth engagement” means operationally in the context of this study. The composite score captures five distinct but conceptually related dimensions of patient interaction with the VSMART application. A patient with a higher engagement score is one who, relative to patients with lower scores, (1) used the application more frequently during treatment (Domain A—Intensity of use); (2) found the application easier to use (Domain B—Ease and acceptability); (3) made greater functional use of the application—including using it to communicate with healthcare providers, accessing health information and educational content, and reporting that the application helped them better understand their disease and feel more in control of their care (Domain C—Functional engagement); (4) experienced fewer disruptions to continuity of use, such as shorter periods of non-use and fewer reinstallations required (Domain D—Continuity); and (5) reported fewer barriers to sustained engagement, specifically fewer reasons for discontinuing application use before treatment completion (Domain E—Discontinuation barriers). “Higher engagement” therefore does not refer to any single dimension in isolation. It reflects a composite of greater behavioural activity, more positive subjective experience, more functional interaction with application features, and fewer disruptions to sustained use. This multidimensional characterisation helps explain why mHealth engagement was associated with both cognitive outcomes (knowledge) and motivational outcomes (attitudes): the functional and experiential dimensions of engagement—particularly perceived benefit for disease understanding and for feeling supported in care—may directly reinforce both informational uptake and treatment-related attitudes.

A key finding of this study was the positive association between mHealth engagement and knowledge. Patients with higher engagement scores had better knowledge scores, and this gradient was clearly observed across engagement tertiles. This is plausible given the role of mHealth applications in reinforcing treatment-related information, reminding patients about medication schedules, and providing accessible guidance on disease management [8,10]. For patients with MDR-TB, who often undergo prolonged and demanding treatment regimens, repeated access to clear and structured information may improve understanding of treatment importance, medication use, and recognition of treatment-related issues, including adverse events.

A similarly strong relationship was observed for attitudes. Patients with greater positive engagement with the application had more favorable attitudes, which may reflect increased confidence in treatment, greater trust in the usefulness of the digital tool, and stronger perceived value of staying connected to care [4]. In the context of MDR-TB, attitudes are highly relevant because treatment is lengthy, burdensome, and frequently accompanied by uncertainty, fatigue, and treatment-related discomfort. An mHealth application may therefore contribute not only to knowledge acquisition, but also to motivation, reassurance, and the perception that treatment is manageable with support [16,17,18].

In contrast, practice was not significantly associated with positive mHealth engagement. This finding is noteworthy and should not be interpreted as meaning that the application lacks value. Rather, it suggests that behavioral practice in MDR-TB care is shaped by a broader set of influences that may not be fully modifiable through digital engagement alone. While an application may improve awareness and attitudes, actual practice related to adherence and response to adverse events may still be constrained by medication side effects, treatment fatigue, social stigma, limited family support, financial barriers, competing life demands, or difficulties accessing health services. In this sense, the absence of a significant association with practice may reflect the complexity of behavioral implementation rather than the absence of benefit from mHealth engagement [19,20].

On the other hand, the absence of an association between mHealth engagement and practice warrants deeper examination. According to the COM-B (Capability–Opportunity–Motivation–Behaviour) model of behaviour change [21], sustained behavioural change requires not only cognitive readiness (Capability) and motivational alignment (Motivation), but also sufficient Opportunity—encompassing the structural, social, and environmental conditions that enable a person to act. The present findings suggest that mHealth engagement may be effective at strengthening Capability—through information delivery and disease education—and Motivation—through perceived support, confidence reinforcement, and positive care relationships. However, Opportunity—the structural dimension of behaviour change—encompasses factors that lie beyond the reach of a digital application: the physical availability of medications, the accessibility of clinic appointments, financial resources, household support, freedom from stigma, and the capacity of the health system to respond to side-effect reports in a timely and supportive manner. In a population of MDR-TB patients experiencing a prolonged and burdensome treatment course with frequent adverse effects, these structural constraints may be the binding determinants of practice, regardless of knowledge or attitudinal improvement. These findings suggest that mHealth may be most appropriately positioned as a tool for improving informational and attitudinal readiness—a necessary but not sufficient condition for practice change—and that its impact on behaviour is likely to be amplified when integrated with strengthened clinical and social support systems.

The present findings are interpretable within the KAP framework, but should not be taken to imply that knowledge, attitudes, and practice form a simple linear causal hierarchy. The assumption that knowledge changes lead to attitude changes, which in turn lead to practice changes, is a theoretical simplification that does not hold universally and has been critiqued on both theoretical and empirical grounds [22,23]. In MDR-TB care specifically, the relationship between these domains is likely non-linear and context-dependent. Patients may develop positive treatment attitudes independently of formal knowledge acquisition—for instance, through social support, trust in healthcare providers, or prior treatment experience—and may maintain or improve their practice regardless of attitudinal change if structural supports such as directly observed therapy or medication delivery are in place. Conversely, patients with high knowledge scores may still show poor practice if treatment side effects are severe, if financial barriers prevent clinic attendance, or if social stigma discourages help-seeking behaviour. The present finding that mHealth engagement was associated with knowledge and attitudes but not with practice is consistent with this non-linear interpretation: the application appears to strengthen the informational and motivational dimensions of patient readiness, but these gains do not automatically translate into behavioural change. Practice in MDR-TB care likely requires not only attitudinal readiness but also structural opportunity—access to medication, clinical support, social enablers—which digital engagement alone cannot provide.

From a programmatic perspective, these findings have several implications for tuberculosis programme design and digital health implementation. First, national tuberculosis programmes—including the Vietnam National Tuberculosis Programme, which has been actively piloting mHealth tools in MDR-TB care—should consider incorporating patient-reported engagement monitoring as a routine component of digital health implementation. The engagement score developed in this study provides a practical model for such monitoring, with demonstrated internal consistency and structural validity. Patients with low engagement scores may warrant additional support, including technical assistance with application use, counselling on the perceived value of digital interaction, or referral to face-to-face support services. Second, consistent with the WHO End TB Strategy’s emphasis on patient-centred care and the integration of digital health tools within broader support systems, these findings caution against positioning mHealth as a stand-alone adherence intervention. The lack of association with practice suggests that the pathway from digital engagement to behavioural change requires structural enablers—counselling, adverse event management, social support, and health system strengthening—that digital tools alone cannot provide. Third, future implementation research should test whether combining high engagement with intensive clinical and social support produces stronger and more sustained behavioural effects than either component alone.

Several limitations should be acknowledged. First, the cross-sectional nature of the analysis limits causal inference. It cannot be determined whether stronger engagement led to better knowledge and attitudes, or whether patients with better knowledge and more positive attitudes were more likely to engage actively with the application [24,25].

Second, 47 participants (16.9%) were excluded from the psychometric and regression analyses due to missing engagement data, concentrated primarily in the conditional discontinuation items (Thongtin11.1–11.4), which were only applicable to patients who had stopped using the application before treatment completion. Although baseline characteristics of included and excluded participants were broadly similar, some selection bias cannot be excluded. Third, KAP outcomes relied on self-report and may be subject to social desirability bias, particularly for practice-related items. Fourth, the multivariable models adjusted for age, sex, and occupation, but did not account for additional potential confounders, including education level, socioeconomic status, disease duration, prior TB treatment history, digital literacy, smartphone ownership, internet access quality, and existing adherence support structures; all of these may independently influence both mHealth engagement and KAP outcomes, and their omission represents residual confounding that may have affected the magnitude of observed associations. Fifth, the presence of two additional PCA components with eigenvalues greater than 1 indicates some degree of multidimensionality in the engagement scale; future confirmatory factor analysis in independent MDR-TB samples would strengthen confidence in the scale’s latent structure and broader applicability. Sixth, the study population consisted specifically of MDR-TB patients enrolled in the V-SMART intervention in Vietnam, limiting generalisability to other TB populations, patients not using digital health tools, or other health system contexts.

Despite these limitations, the study has notable strengths. It addresses a highly relevant topic in TB care by linking digital engagement with patient-centered outcomes in a population facing prolonged and complex treatment. The analysis also proceeds in a coherent sequence, from psychometric assessment of the engagement scale to examination of its associations with KAP outcomes and comparison across tertiles. In addition, the separation of knowledge, attitudes, and practices provides a more nuanced interpretation than would be possible from a single combined outcome alone. This is especially valuable in MDR-TB care, where improvement in knowledge or attitudes may represent meaningful progress even before measurable changes in behavior are observed.

In summary, among MDR-TB patients using an mHealth application for treatment support and adverse event management, higher positive mHealth engagement was associated with better knowledge and more favorable attitudes, but not with significantly different practice scores. The positive mHealth engagement scale also demonstrated good reliability and acceptable psychometric performance in this study population. These findings suggest that mHealth may be a useful tool for strengthening informational and attitudinal readiness in MDR-TB care, but that behavioral practice likely depends on broader structural, clinical, and social factors. Future research should assess these relationships longitudinally and examine whether integrating mHealth engagement with more intensive adherence and adverse event support can lead to stronger behavioral effects.

## 5. Conclusions

Among MDR-TB patients using a smartphone-based mHealth application for treatment support and adverse event reporting, higher positive mHealth engagement was significantly associated with better knowledge and more favourable treatment-related attitudes, while no significant association was observed with practice. These findings are associative rather than causal; the cross-sectional design does not permit conclusions about the direction of the observed relationships. The positive mHealth engagement scale demonstrated good internal consistency (Cronbach’s α = 0.864 for the refined 11-item score) and acceptable structural validity, supporting its use as a summary measure of patient interaction with mHealth interventions in MDR-TB care.

From a public health perspective, these findings support the integration of mHealth tools within MDR-TB care as a means of strengthening patient knowledge and treatment-related attitudes—components of patient readiness that are necessary, though not sufficient, for sustained practice change. National tuberculosis programmes should consider incorporating engagement monitoring into routine digital health implementation to identify patients who may require additional support. At the same time, the absence of an association with practice underscores the need for integrated strategies that combine digital engagement with face-to-face counselling, adverse event management, social support, and health system strengthening. Consistent with the WHO End TB Strategy, mHealth should be positioned as a complementary tool within a comprehensive patient-centred care framework, not as a stand-alone intervention. Future longitudinal research should evaluate whether improvements in knowledge and attitudes mediated by mHealth engagement translate into practice change when embedded within such integrated support structures.

## Figures and Tables

**Table 1:** Descriptive statistics of KAP outcomes and positive mHealth engagement.

Variable	n	Mean ± SD	Median (IQR)	Min–Max
Knowledge score	278	9.95 ± 3.39	11.0 (8.0–13.0)	0–16
Attitude score	278	35.63 ± 5.99	35.5 (31.0–41.0)	22–44
Practice score	278	18.25 ± 2.37	19.0 (18.0–20.0)	8–21
Total KAP score	278	63.83 ± 8.40	64.0 (57.0–71.0)	42–78
Positive mHealth engagement score	231	0.11 ± 0.50	0.03 (−0.31–0.55)	−1.39–1.47

**Table 2:** Psychometric properties of the positive mHealth engagement scale. KMO, Kaiser–Meyer–Olkin measure of sampling adequacy. Bartlett’s test was significant, indicating that the inter-item correlation matrix was suitable for dimensionality assessment.

Measure	Value
Number of items	12
Complete cases used for analysis	231
Cronbach’s alpha	0.852
Kaiser–Meyer–Olkin (KMO)	0.870
Bartlett’s test of sphericity, χ^2^	1351.31
Degrees of freedom	66
Bartlett’s test *p*-value	<0.001

**Table 3:** Association between positive mHealth engagement and knowledge, attitude, practice, and total KAP scores.

Outcome	β	β* (Standardised)	95% CI	*p*-Value
Knowledge score	2.06	0.304	1.28 to 2.85	<0.001
Attitude score	4.68	0.391	3.30 to 6.06	<0.001
Practice score	−0.07	−0.015	−0.63 to 0.49	0.804
Total KAP score	6.68	0.398	4.62 to 8.74	<0.001

β* = β × (SD engagement/SD outcome) = β × (0.50/SD_Y).

**Table 4:** Comparison of KAP scores across tertiles of positive mHealth engagement.

Outcome	Low Tertile	Moderate Tertile	High Tertile	*p*-Value
Knowledge score	8.42	10.67	11.69	<0.001
Attitude score	32.53	35.67	39.21	<0.001
Practice score	18.42	18.33	18.23	0.847
Total KAP score	59.37	64.67	69.13	<0.001

## Data Availability

The data presented in this study are available on request from the corresponding author. The data are not publicly available due to privacy and ethical restrictions, as the dataset contains sensitive information related to patients within the National Tuberculosis Program and is subject to the data protection policies of the collaborating institutions.
